# WAVES – The Lucile Packard Children’s Hospital Pediatric Physiological Waveforms Dataset

**DOI:** 10.1038/s41597-023-02037-x

**Published:** 2023-03-07

**Authors:** Daniel R. Miller, Gurpreet S. Dhillon, Nicholas Bambos, Andrew Y. Shin, David Scheinker

**Affiliations:** 1 Stanford University; Department of Electrical Engineering, Palo Alto, CA 94304 USA; 2 Stanford University School of Medicine; Lucile Packard Children’s Hospital at Stanford; Department of Pediatrics, Division of Pediatric Cardiology, Palo Alto, CA 94304 USA; 3 Stanford University; Department of Management Science and Engineering, Palo Alto, CA 94304 USA; 4 Stanford University; Clinical Excellence Research Center, Palo Alto, CA 94304 USA; 5 Stanford University School of Medicine; Lucile Packard Children’s Hospital at Stanford; Department of Pediatrics, Division of Pediatric Endocrinology, Palo Alto, CA 94304 USA

**Keywords:** Health care, Paediatric research

## Abstract

WAVES is a large, single-center dataset comprising 9 years of high-frequency physiological waveform data from patients in intensive and acute care units at a large academic, pediatric medical center. The data comprise approximately 10.6 million hours of 1 to 20 concurrent waveforms over approximately 50,364 distinct patient encounters. The data have been de-identified, cleaned, and organized to facilitate research. Initial analyses demonstrate the potential of the data for clinical applications such as non-invasive blood pressure monitoring and methodological applications such as waveform-agnostic data imputation. WAVES is the largest pediatric-focused and second largest physiological waveform dataset available for research.

## Background & Summary

High-frequency physiological waveform data are commonly used to monitor patient cardiac, circulatory, and respiratory status. The availability of adult clinical and physiological waveform data, much of it in research repositories, has had a profound impact on the research community, leading to numerous clinical and methodological advances^1–13^. However, pediatric patients differ from adult patients to such a degree that models developed for and trained on adult data do not generalize well to pediatrics. For example, a resting heart rate of 150 is normal for a newborn and considered tachycardia in adults. Currently, no large repositories of high-frequency pediatric physiological waveform data are widely available for research. The creation of such a repository and the tools to analyze it are critical to enable developments in pediatric medicine with machine learning (ML).

The latest iteration of the Medical Information Mart for Intensive Care (MIMIC-III) is a freely accessible critical care database which includes physiological waveforms, vital signs, laboratory measurements, survival data, and more^3^. These data have provided the basis for almost two decades of annual Computing in Cardiology ML competitions through the PhysioNet organization as well as collaborative efforts and code-sharing amongst researchers to drive progress in the fields of ML and medicine^12^. The UK Biobank is an open-access database with the specific goal of identifying the causes of a complex diseases of middle and old age^13^. The ability of researchers to access these data has facilitated numerous analyses towards diverse research goals.

Architectures leveraging advances in deep neural networks have demonstrated success using large volumes of unprocessed (i.e., unstructured) data such as physiological waveforms and medical images^4,7–11^. Medical research datasets have enabled a generation of research efforts in medical ML and deep learning^3,12,13^. Similar pediatric-focused research datasets are neither easily nor widely available and models and conclusions based on adult data cannot be generalized to pediatric populations. Leveraging the power of ML to improve the quality of pediatric care requires large, well-organized, pediatric-specific open-access datasets to be made available for research purposes.

**Fig. 1:**
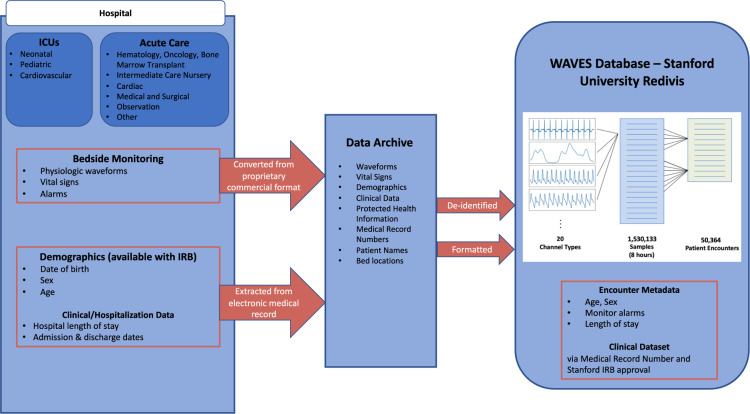
Overview of WAVES Database. From left to right: Intensive care units and acute care floor units were included in this dataset. Bedside monitoring data, demographic data, and clinical/hospitalization data was collected, converted, formatted, and placed into a data archive which was then de-identified. Refined physiologic waveform and vital sign data were then uploaded to Stanford University Redivis as the Pediatric WAVES dataset.

WAVES is a large, single-center dataset comprising 9 years of high-frequency physiological waveform data from patients in intensive and acute care units at a large academic, pediatric medical center (Fig. 1) that has been deposited to https://redivis.com/datasets/heph-f0yqqyy64^14^. Researchers can register an account and obtain access to the WAVES dataset at https://redivis.com/WAVES/datasets. WAVES consists of 10.6 million hours of 1 to 20 concurrent types of high-frequency physiological waveforms. Approximately 1.5 million waveform samples were collected over 50,364 encounters, with each encounter defined as all units visited during one hospitalization. Patient date of birth was recorded for 40.5% of encounters and patient sex was recorded for 53.8% of encounters. For those encounters for which it was available, median age at start of the first waveform measurement was 4.2 years (interquartile range 94 days to 11.12 years) and patients were under the age of 18 at the start of 95.8% of encounters. Sex was female for 54.3% of encounters where male/female data on patient sex was recorded (total numbers: 46.1% male, 38.8% female, 15.1% unidentified/refused to answer/non-male and non-female identifying). WAVES is currently the largest pediatric-focused physiological waveform dataset available for research and the second largest repository of correlated multi-channel physiological waveform data (second to the MIMIC-III clinical database^3^). The availability of parallel timeseries with various degrees of correlation and readily interpretable features, e.g., low blood pressure, may be a valuable resource for methodological time series research^15–17^.

The objective of WAVES is to enable improvements to pediatric clinical care through ML research on rich physiological data from a variety of hospitalized pediatric patients. Such research will include training unsupervised models for data processing, imputation, and the identification of relationships between physiological signals. These data will also facilitate work developing supervised models to identify or predict clinical events, monitor difficult-to-observe aspects of patient health state, and track changes in patient health. Initial analyses demonstrate the potential of WAVES for clinical applications such as non-invasive blood pressure monitoring and methodological applications such as waveform-agnostic missing data imputation^4,18^. Further use of this large, rich data set will facilitate the development of methodological and clinical innovation in the field of pediatric care.

## Methods

### Setting

Systems Utilization Research for Stanford Medicine (SURF Stanford Medicine) is an interdisciplinary collaboration of engineers, practicing physicians, and members of university and hospital information services. SURF has extensive experience in data-driven healthcare modeling^3,18–20^.

### Database development

The Stanford WAVES dataset contains over 10.6 million hours of concurrent physiologic waveforms associated with 50,364 unique encounter ids as well as vital sign and demographic data recorded from patients hospitalized at a pediatric academic medical center between June 2008 and January 2017. The physiologic waveforms collected in this dataset include: electrocardiogram (ECG), respiratory, plethysmography, arterial blood pressure (ABP), end-tidal carbon dioxide level (etCO2), central venous pressure (CVP), pulmonary arterial pressure (PAP), umbilical vein pressure (UVP), umbilical arterial pressure (UAP), and right atrial pressure (RAP) (Table 1). Vital signs collected in this dataset include non-invasive blood pressure (NBP) (systolic, diastolic, and mean), arterial blood pressure (systolic, diastolic, and mean), heart rate, pulse rate, respiratory rate, temperature (core, esophageal, core, rectal, skin), peripheral capillary oxygen saturation (SpO2), etCO2, cerebral perfusion pressure, pulmonary arterial pressure (systolic, diastolic, and mean), umbilical arterial pressure (systolic, diastolic, and mean), electrocardiogram (ECG) ST segments, and ECG QT and QTc intervals (Table 2). Vital signs are recorded once per minute and the WAVES dataset contains 117.2 million minutes of vital sign data records over all patients/encounters.

**Table 1:** Sample Counts and Total Duration in Hours by Waveform Type.

Waveform Type	Samples	Total Hours
Electrocardiogram (ECG)	550,280	3,818,717
Plethysmography (PLETH)	510,771	3,545,108
Respirations (RESP)	413,701	2,868,872
Arterial Blood Pressure (ABP)	32,709	251,707
End-tidal CO2 (etCO_2_)	6,664	51,457
Central Venous Pressure (CVP)	3,477	27,780
Plethysmography (PLETH) - Left	6,234	37,438
Plethysmography (PLETH) - Right	3,532	21,914
Plethysmography (PLETH) – Preductal	213	1,395
Plethysmography (PLETH) – Postductal	103	616
Plethysmography (PLETH) – Telemetry	609	4,454
Pulmonary Arterial Pressure (PAP)	262	2,042
Umbilical Vein Pressure (UVP)	149	1,065
Umbilical Arterial Pressure (UAP)	57	415
Right Atrial Pressure (RAP)	33	258

**Table 2:** Vital Signs.

Vital Sign	Total Hours
RESP	1,599,240
HR	1,618,624
PULSE	1,636,026
PULSE_NBP	1,275,045
SpO2	1,584,401
NBP_SYS	1,662,726
NBP_DIAS	1,662,726
NBP_MEAN	1,662,726
ABP_SYS	139,311
ABP_DIAS	139,311
ABP_MEAN	139,311
etCO2	141,406
CVP	139,808
TEMP	21,616
TEMP_RECTAL	36,118

RESP – respiratory rate per minute; HR – heart rate in beats per minute; PULSE - heart rate in beats per minute; PULSE_NBP - heart rate in beats per minute as recorded from non-invasive blood pressure monitor; SpO2 – oxygen saturation determined by plethysmography; NBP_SYS – systolic blood pressure by non-invasive methods; NBP_DIAS – diastolic blood pressure by non-invasive methods; NBP_MEAN – mean blood pressure by non-invasive methods; ABP_SYS - systolic blood pressure by invasive arterial monitoring; ABP_DIAS - diastolic blood pressure by invasive arterial monitoring; ABP_MEAN = mean blood pressure by invasive arterial monitoring; etCO2 – end-tidal carbon dioxide level (invasive or non-invasive); CVP – central venous pressure (invasive); TEMP- body temperature; TEMP_RECTAL – body temperature through rectal monitoring.

### Dataset creation

Physiologic waveforms, vital signs, demographic data, and clinical data were downloaded from bedside patient monitoring systems and hospital electronic health records. Waveform data from bedside monitors were uploaded and codified via Philips Information Systems bedside monitors (Series MP 5/30/50/70/90 & Series MX 40/400450/500/800). The WAVES dataset is stored in Redivis (Redivis, Stanford University, Mountain View, CA), a web-based data platform for researchers and data administrators with the ability to compute and analyze data, managed by the Stanford Center for Population Health Sciences. The Redivis WAVES dataset can be accessed via https://redivis.com/WAVES/datasets^14^. Linking of physiologic and vital sign variables to clinical data for research purposes is possible through the Stanford Healthcare Box Database and the Stanford Research Repository (STARR) after Stanford Institutional Review Board (IRB) approval. Physiologic data are compiled, cleaned, and maintained through the Department of Management Science and Engineering.

### Data processing and cleaning

All bedside monitoring system data initially contained protected health information (PHI), including: name, bed location, date, medical record number (MRN), and other identifiers which were stored on secure drives at Lucile Packard Children’s Hospital (LPCH). Philips monitoring data was used to extract waveforms into scalable open-source format, which was then deidentified. All Philips monitoring data were formatted with proprietary software (Philips IntelliVue system) which compresses and saves the raw waveforms into a proprietary format. The Philips proprietary RDE2WAV software was used to extract the waveforms into a scalable open-source format (HDF5) as part of the de-identification process. Python code was written to perform the extract/transform/load (ETL) process, which included: 1) transferring the file from storage to a secure and encrypted machine running the ETL code, 2) extracting the raw data from the file, 3) cleaning and de-identifying the data, 4) organizing and packaging the data into HDF5, including recompression, and 5) pushing the HDF5 file to secure cloud storage. Data that were converted to standard compressed de-identified HDF5 files (which are indexed) can be easily searched, grouped, and can be read by open-access R and Python analytical software packages.

Patient names, bed location, and MRN data were removed, and date shifting was performed on waveform data in accordance with Health Insurance Portability and Accountability Act (HIPAA) standards in order to de-identify the data. All encounters were shifted to a zero date of January 1^st^, 2000. Furthermore, all data within a single encounter was aligned to maintain the original relative positions and times-of-day within that encounter, even if the actual dates of the encounter were being changed. Thus, all patient hospitalization timelines are maintained in the wave id time shifts. For example, if waveform timelines are dated from 07/01/2012 to 08/01/2012, these dates were shifted to 01/01/2000 to 02/01/2000 to maintain relative positions within a hospitalization.

## Data Records

WAVES is hosted in the Redivis data repository system as a SQL-accessible system backed by Google BigQuery^14^. WAVES consist of three primary tables: waveforms, dates, and vitals. Tables are linked by a “wave_id” identifier, which is roughly analogous to a single hospital encounter for an individual patient (with possible splits based on intra-hospital departmental transfers). Within each wave_id record, “group” identifiers are used to split the records into contiguous time-windows of at most 8 hours, determined by the hardware limitations of the original bedside monitor systems (Table 3).

**Table 3:** An Overview of the WAVES tables.

Table	Frequency	Description
Waveforms	125/sec	Actual physiological waveforms and metadata organized into roughly 8-hour chunks
Vitals	1/min	Calculated vital signs (e.g., heart rate, oxygen saturation)
Dates	1/sec	De-identified dates for the waveform arrays that can be used to match/join the waveforms with vital sign data

Each table is accessible and documented within Redivis, and may be queried, filtered, joined, along with a variety of other standard query operations before downloading extracts as .csv files.

Since the raw waveform arrays are extremely large, it would be storage-inefficient to include the timestamps for every individual sample. Instead, each waveform sample is stored with start and end indices and datetimes, and a constant sample rate/frequency. In addition, the *dates* table can be used to find specific reference indices within the waveform corresponding to specific dates/times. This also allows for validation and correction for anomalous breaks in the record corresponding to events like temporary monitor disconnection.

The vitals table includes 69 different types of vital signs. These range from very standard observations recorded for most records, such as heart rate (HR), oxygen saturation (SPO2), respiratory rate (RESP), and temperature (TEMP) to more specialized observations which are more sparsely populated in the dataset, such as intracranial pressure (ICP) or the change in QT interval (DELTA_QTC) (Table 2).

Lucille Packard Children’s Hospital houses multiple varied critical care units and floor acute care units that admit patients based on severity of illness and medical indication for hospital admission. The various units from which waveform and demographic data were gathered provide care for distinct pediatric patient populations, including: neonatal, pediatric, and cardiovascular intensive care units; cardiac, hematology/oncology/bone marrow transplant, and medical and surgical acute care units; and an observational unit and intermediate care nursery (Table 4).

**Table 4:** Waveform Demographics.

Hospital Department/Unit	Number of Distinct Encounters With Age/Sex Availability	Median Age (25^th^,75^th^), in days	Female (%)	Total Waveform Hours
**Intermediate Care Nursery**	65,694	9 (3,33)	30,088 (45.8)	30,060,466
**Hematology/Oncology/Bone Marrow Transplant**	4,905	3,330 (1551,5802)	1,928 (39.3)	3,113,972
**Neonatal ICU**	61,428	14 (1,43)	28,827 (46.9)	41,850,629
**Cardiovascular ICU**	1,990	823 (58,3357)	975 (49.0)	1,025,531
**Pediatric ICU**	70,026	1,910 (434,4775)	31,011 (44.3)	31,727,131
**Cardiac Acute Care**	3,290	3,976 (865,4012)	1,971 (59.9)	6,236,823
**Medical Acute Care**	10,150	4,413 (1531,5903)	5,905 (58.2)	3,633,045
**Medical/Surgical Acute Care**	11,917	3,818 (1347,5095)	5,645 (47.4)	7,119,761
**Surgical Acute Care**	21,076	2,975 (1061,5177)	9,703 (46.0)	4,298,047
**Transplant (Solid Organ) Acute Care**	10,726	3,137 (822,5035)	5,330 (49.7)	3,546,838
**Observation**	110	3,594 (2024,6184)	50 (45.5)	7,110
**Unknown/Other**	650	2,584 (295,4558)	230 (35.4)	235,085

ICU – intensive care unit

Unit-level statistics are approximations based on the majority of the data that included patient and unit metadata.

Department/Unit patient type descriptors: Intermediate Care Nursery - Neonates/Infants requiring ongoing inpatient care with support needs below level needed for NICU care; Hematology/Oncology/Bone Marrow Transplant - Patients with underlying hematologic/oncologic illness processes requiring admission on the acute care floor; Neonatal ICU - Critically Ill Neonates/Infants requiring ICU level of care; Cardiovascular ICU - Critically ill children of all age ranges with heart disease requiring ICU level of care; Pediatric ICU - Critically ill children of all age ranges requiring ICU level of care (majority of who do not have underlying heart disease); Cardiac Acute Care - Children of all ages with heart disease requiring admission to the hospital not requiring ICU level of care; Medical Acute Care – Children of all ages with medical illnesses requiring admission to the hospital not requiring ICU level of care; Medical/Surgical Acute Care - Children of all ages with medical and/or surgical disease processes requiring admission but not ICU level of care; Surgical Acute Care - Children of all ages with surgically-managed disease processes requiring admission but not ICU level of care; Transplant - Children of all ages with disease processes related to organ transplantation requiring admission but not ICU level of care; Observation - Patients of all ages admitted for observation for periods <24 h from the Emergency Department for monitoring; Unknown/Other - Patients admitted to the hospital under unspecified units/services.

The vitals and waveform tables may be joined using the wave_id, group, and date/time columns. However, since they are recorded at different sample rates, any predictive modeling should be carefully considered and designed to account for this data structure. In many cases, such as when using convolutional neural network models, it may be most suitable to query and load each table separately and join within the model itself or via ensemble methods.

## Technical Validation

The WAVES dataset closely follows the data schema of the original data collected within Lucile Packard Children’s Hospital. Changes to the structure were restricted to those necessary for de-identification, aggregation, and conversion to openly accessible compression and file formats. Loss and consolidation of data from the original source was minimized to only that necessary for de-identification.

The code used to build WAVES was version-controlled and tested before use. The primary extract/transform/load (ETL) code has been shared with external collaborators to facilitate access to the raw source data among the wider research community.

Although this is the first public release of the WAVES dataset and there has not yet been an opportunity for community review and feedback, all users are encouraged to report issues. The WAVES project helper and utility codebase is provided as an open-source repository to encourage collaboration, transparency, and feedback.

## Usage Notes

### Data access

Vital sign and waveform data can be imported via any standard programming language and examples are provided for both Python and R in our open-source repository. Queried and filtered data from Redivis can be downloaded into CSV format via various statistical software platforms. The raw waveform data is included in the CSV files as a base-64 encoded string.

All researchers can formally request data via Redivis, and detailed information regarding access requests can be found on the Redivis website (https://redivis.com/for-researchers). Part of the request-for-access process for researchers includes Collaborative Institutional Training Initiative (CITI Program) training in accordance with HIPAA compliance as the data deals with human research participants. Alongside HIPAA compliance training, signed data user and non-disclosure agreements are required by researchers. Once compliance training is completed and any necessary IRB approval has been received, the researcher will receive instructions from Redivis containing detailed instructions on how to access and download the WAVES dataset as CSV files. Examples of these downloading instructions can be found at https://bitbucket.org/surfstanfordmedicine/waves-utilities/src/main/ and https://pypi.org/project/waves-utilities/. Standard approved users will be given access to a 1% random sample, while full access will require specific approval from the SURF team and Stanford IRB. This 1% standard access allows for the ability to build cross-validation sets for fair competitions for users by restricting access to potential test/validation data. Although we have found that R and Python can readily process and analyze WAVES CSV files, all data are in openly defined data formats and encodings and any programming language can be used to read and manipulate the WAVES data. In order to obtain demographic data and link it to vital sign and waveform ids to enable re-identification and combined analysis with other clinical data sources, the researcher will be required to obtain Stanford University IRB approval with a Stanford faculty researcher serving as primary investigator for the IRB.

Additional information regarding instructions on downloading CSV files, scripts on converting CSV files to programming language, dataloaders, plotting examples, a fully-documented application programming interface (API), pytests, and information on plotting sample waveforms can be found at https://pypi.org/project/waves-utilities/ and https://bitbucket.org/surfstanfordmedicine/waves-utilities/src/main/.

### Example usage

The pediatric WAVES data has been downloaded and analyzed to identify a hypotensive state as measured with an arterial catheter, using data from multiple noninvasive sensors^4^. In a model utilizing convolutional-deconvolutional networks, a real-time probability estimate of hypotension was created using non-invasively obtained waveforms. In that study, Miller *et al*. depict the structure of the convolutional-deconvolutional network, use of training, validation, and test sets, and AUPRC validation to show that non-invasive waveforms can be used to replicate invasive arterial blood pressure monitoring for continuous identification of arterial hypotension using data from noninvasive sensors.

The WAVES dataset has also been utilized to demonstrate how deep learning techniques can reconstruct missing data using patient-specific patterns present in the non-missing portions of the waveform. Using a convolutional neural network trained on waveform samples, the WAVES dataset has been successfully used to develop a generalizable model to analyze and extract information from arbitrary physiological waveforms and used to develop methods for mid-channel missing time-series imputation^18^. A demonstration datafile of waveforms can also be downloaded by researchers who wish to sample and evaluate WAVES data without first having to register through Redivis, at https://redivis.com/datasets/heph-f0yqqyy64^14^.

### Collaborative research

Our goal in compiling the pediatric WAVES dataset is to promote an open-source repository of physiologic and vital sign data to allow researchers to collaborate, download data, and share code that allows for the study of physiologic states of hospitalized pediatric patients to identify, predict, and assist in the treatment of pediatric medical conditions. Our git repository (https://bitbucket.org/surfstanfordmedicine/waves-utilities) contains data loading instructions regarding linking to Redivis, instructions on downloading CSV files, waveform visualization, performing vital sign statistics and cohort selection, and provides scripts on how to convert CSV files to programming language. The repository also contains waveform visualization examples (e.g., plotting respiratory, blood pressure, or ECG waveforms) as well as directions on how to calculate aggregated vital sign statistics (e.g., maximum, minimum, mean, median values). Analysis of vital signs can allow a researcher to identify certain trends, such as heart rate or respiratory rate variability during a pediatric admission, or maximum/minimum heart rates and respiratory rates for a specific population of admitted pediatric patients. The database allows for cohort selection based on the clinical question being asked by the researcher (e.g., only male patients under five years of age admitted to the cardiovascular intensive care unit with physiologic waveform samples that contained greater than 15 minutes of plethysmography data). For example, a study evaluating pediatric hypotension using non-invasive parameters in the pediatric WAVES dataset^4^ restricted to samples that contained at least 15 minutes of arterial blood pressure (ABP) waveform data. IRB approval allows for linking of physiologic and vital sign data with patient data from the electronic medical record, including: diagnoses, medications, procedures, etc. WAVES also allows table-joining, which can combine both physiologic waveform and vital sign samples from a unique patient encounter (e.g., combining plethysmography waveform with heart rate/respiratory rate/SpO2 vital sign data). This could potentially strengthen statistical analyses and validate physiologic waveform and vital sign data based on the degree of correlation between the two combined datasets.

We provide the opportunity and encourage researchers using the WAVES data to contribute to the open-source repository with new code, updated instructions, and feedback which could benefit future research utilizing this database.

## Data Availability

Redivis provides a visual drag-and-drop filtering user interface that allows the user to select columns of interest, filter on properties of interest, and limit output parameters before creating a downloadable CSV file. Sample scripts for working with data downloaded from Redivis and plotting sample waveforms are available in open-source repositories: https://bitbucket.org/surfstanfordmedicine/waves-utilities/src/main/ and https://pypi.org/project/waves-utilities/.
